# Bacterial Communities and Prediction of Microbial Metabolic Pathway in Rice Wine Koji From Different Regions in China

**DOI:** 10.3389/fmicb.2021.748779

**Published:** 2022-01-03

**Authors:** Xinxin Zhao, Fanshu Xiang, Fengxian Tang, Wenchao Cai, Zhuang Guo, Qiangchuan Hou, Xinquan Yang, Wen Song, Chunhui Shan

**Affiliations:** ^1^School of Food Science, Shihezi University, Shihezi, China; ^2^Engineering Research Center for Storage and Processing of Xinjiang Characteristic Fruits and Vegetables, Ministry of Education, Shihezi University, Shihezi, China; ^3^Hubei Provincial Engineering and Technology Research Center for Food Ingredients, Hubei University of Arts and Sciences, Xiangyang, China

**Keywords:** bacterial diversity, Illumina Miseq high-throughput sequencing, rice wine koji, lactic acid bacteria, functional prediction, phenotypic prediction

## Abstract

Rice wine koji, a traditional homemade starter culture in China, is nutritious and delicious. The final quality of rice wine koji is closely related to the structure of its microbial community. However, the diversity of natural microorganisms in rice wine koji from different regions has not been evaluated. In this study, the microbial population of 92 naturally fermented rice koji samples collected from Hubei, Guangxi, and Sichuan was systematically analyzed by high-throughput sequencing. From all the rice wine koji samples, 22 phyla and 479 bacterial genera were identified. *Weissella*, *Pediococcus*, *Lactobacillus*, *Enterobacter*, *Lactococcus*, *Pantoea*, *Bacillus*, *Staphylococcus*, and *Leuconostoc* were the dominant genera in rice wine koji. The bacterial community structure of rice wine koji samples from different regions was significantly different (*p* < 0.05). The bacterial community composition of the samples from Hubei and Guangxi was similar, but significantly different from that of SC samples (*p* < 0.05). These differences may be caused by variations in geography, environment, or manufacturing. In addition, the results of microbial phenotype prediction by BugBase and bacterial functional potential prediction by PICRUSt showed that eight of the nine predicted phenotypic functions of rice wine koji samples from different regions were significantly different (*p* < 0.05) and that vigorous bacterial metabolism occurred in rice wine koji samples.

## Introduction

Koji is produced by solid-state anaerobic fermentation in a specific ecological environment, using mature koji as the parent strain, and contains many microorganisms, including bacteria and fungi, which play a decisive role in the quality of rice wine. The Hubei (HB), Sichuan (SC), and Guangxi (GX) provinces have characteristic geographical features, and the local residents have a wealth of experience in producing various fermented foods using native environmental microorganisms. As a traditional starter culture, rice wine koji offers an excellent model to study the diversity of environmental microorganisms and the regional sources of beneficial microorganisms (El Sheikha, 2018). Hui et al. (2017) used single-molecule, real-time sequencing technology (SMRT) to identify a large number of bacteria belonging to *Ochrobactrum* in three Japanese rice wine koji. Bora et al. (2016) used a whole-genome shotgun sequencing method based on the Illumina platform to study the rice wine koji, Xaj-pitha, in India and found that rice wine koji contained a large number of bacteria genera with lactic acid bacteria (LAB) as the main bacterial group. Because fermented food is made by traditional technology and is relatively open to the environment, rice wine koji produced by different fermentation technologies and environmental conditions has unique aroma, flavor, and texture. Therefore, the LAB bacteria resources contained within rice wine koji may be unique and diverse, with a richer and more complex microbial community than previously thought (Ray et al., 2014). Thus, the complexity of the microbial community structure of rice wine koji may be the cause of variations in product quality in most rice wine enterprises. Recent studies (Zhao et al., 2020) have shown that the bacterial community in rice wine koji is composed of several dominant genera belonging to Firmicutes and Proteobacteria, including *Weissella*, *Lactobacillus*, *Lactococcus*, and *Cronobacter*. However, our research focused on the α diversity of microorganisms and the separation of beneficial microorganisms. To perform an extensive study of the similarity and difference in microbial composition among regions, find the distribution diversity of microorganisms determined by geography, and further select and apply beneficial microorganisms, we performed a comprehensive and systematic analysis of rice wine koji samples collected from three major producing areas in China (Hubei, Sichuan, and Guangxi), as determined by previous experiments.

The crucial roles of microorganisms in producing fermented foods have been well acknowledged (El Sheikha et al., 2018). Diversified functional microorganisms in fermented foods can also enhance the desired texture, aroma, and flavor (Dertli and Çon, 2017). It has been widely reported that LAB play a major role in fermentation and an important role in the intrinsic characteristics of fermented products, affecting the development of aroma, texture, and acidity of products (Cai et al., 2019). Previous studies have shown that LAB are equipped with an enzyme system that metabolizes amino acids and can be used for flavor formation in foods such as cheese, wine, and fermented sausages (Sodini et al., 2002).

Pure culture technology leads to bias and limits the research into microbial diversity (El Sheikha and Hu, 2020). The second-generation high-throughput sequencing technology of Illumina MiSeq makes up for the shortcomings of the aforementioned technology, allowing the measurement of millions of DNA molecules at the same time and the analysis of the gene composition and functional characteristics of specific microbial populations based on their gene sequences. It has various advantages: simple operation, low cost, and the good feasibility of results (Liu et al., 2019). This technology has been used for the analysis of the microbial population structure of a wide range of various fermented foods and vegetables, such as kimchi (Luo et al., 2021), rice wine (Cai et al., 2018), and soy sauce (Zhao et al., 2021), and has accelerated our understanding of the microbial diversity of rice wine koji.

In this study, we collected 92 samples of rice wine koji from agricultural produce markets in the HB, SC, and GX provinces of China. By sequencing the V3–V4 region of the bacterial 16S rRNA gene, we analyzed the diversity of the microbial population and the differences among samples by multivariate statistical analysis, performing a thorough evaluation of the complex microbial communities in rice wine koji from different regions. In addition, we aimed to predict the phenotypic changes in the microbial community in rice wine koji samples, and to explore the commonness and differences of samples from three regions from the perspectives of function and bacterial phenotype. Through this study, we hope to achieve a comprehensive analysis of the characteristics of rice wine koji community flora, enhance our understanding of the core microbial community involved in traditional fermentation and its contribution to the fermentation process, and provide strain support and data support for the development of new starter cultures.

## Materials and Methods

### Sample Collection

In this study, a completely random experimental design was used to collect samples from local farmers’ markets (Figure 2A) in Xiaogan city in Hubei province (113.91°E, 31.92°N), Dazhou city in Sichuan province (107.21°E, 30.75°N), and Nanning city in Guangxi province (108.20°E, 22.48°N). The samples were collected, placed in a sterile sampling box containing ice bags, transported back to the laboratory at low temperature, ground into powder in a sterile mortar, and then placed in a freezer at –80°C until use. In accordance with the order of collection, rice wine koji samples from Hubei province were labeled MJQHB1–MJQHB31, rice wine koji samples from Sichuan province were labeled MJQSC1–MJQSC30, and rice wine koji samples from Guangxi province were labeled MLQGX1–MLQGX31.

### DNA Extraction and Target Gene Amplification

In accordance with the manufacturer’s instructions, metagenomic DNA was extracted from 2.0 g of each sample using the QIAGEN DNeasy mericon Food Kit (QIAamp DNA microbiome kit; QIAGEN Inc.). The quality of DNA was analyzed using the OD 260/280 ratio, and agarose gel electrophoresis (1% w/v agarose in 0.5 × tris–acetate–EDTA buffer) was used to detect the concentration, purity, and integrity of the extracted deoxyribonucleic acid. Qualified DNA samples were stored at –20°C until further analysis (Cai et al., 2021a).

The full-length bacterial 16S rRNA gene sequences were amplified from all genomic DNA samples by PCR for barcode sequencing using the forward 338F (5′-ACTCCTACGGGAGGCAGCAG-3′) and the reverse 806R (5′-GGACTACHVGGGTWTCTAAT-3′) primers (Mosher et al., 2013); a set of eight nucleotide barcodes was added to each forward primer to distinguish the source of the sequences (Cai et al., 2021b). The PCR amplification was performed as follows: initial denaturation at 95°C for 3 min, followed by 30 cycles of denaturation at 95°C for 30 s, annealing at 58°C for 30 s, and extension at 72°C for 45 s, complete extension at 72°C for 10 min, and finally at 10°C until halted by user (Zhao et al., 2020). The PCR mixture contained 4 μl 5 × TransStart FastPfu buffer, 2 μl of 2.5 mM dNTPs mixture, 0.8 μl of forward primer (5 μm), 0.8 μl of reverse primer (5 μm), 10 ng of template DNA, 0.4 μl of TransStart FastPfu DNA polymerase, and was made up to 20 μl with ddH_2_O. PCRs were performed in triplicate (Cai et al., 2021c).

### MiSeq High-Throughput Sequencing and Bioinformatics Processing

The amplicon was diluted to 100 nmol/L and then sent to Shanghai Major Bio Co., Ltd. Shanghai, China, for sequencing using the MiSeq high-throughput sequencing platform (Illumina, San Diego, United States).

Based on the overlapping relationship between the paired sequences, the data from the two terminal sequences of the off-board are spliced into one sequence. Before bioinformatics analysis, internal python scripts were used to remove the primers and sequence barcodes, and then all readings are classified into different samples based on their barcodes. The qualified quality control sequences were evaluated for bacterial and microbial diversity and species analysis using the QIIME platform with reference to Caporaso (Caporaso et al., 2010a) and other methods: (1) use PyNAST to align the sequence (Caporaso et al., 2010b); (2) UCLUST is merged under 100% similarity (Edgar, 2010), a single sequence set of 16S rRNA V3–V4 region is established, and then UCLUST is used to classify the unique sequence set into operational taxonomic units (OTUs) under the threshold of 97% identity; (3) use Chimera Slayer to remove OTU sequences containing chimera sequences (Haas et al., 2011); (4) each OTU is assigned to the lowest taxonomic level based on information extracted from Sliva (version 132) (Quast et al., 2012), Greengenes (version 13.8) (DeSantis et al., 2006), and the Ribosomal Database Project (RDP, Release 11.5) (Cole et al., 2007) to determine the physiology and relative foundation of the OTUs at a minimum bootstrap threshold of 80%; (5) use Fast Tree software to generate the phylogenetic tree based on OUT representative sequences (Price et al., 2009); (6) determine the Shannon–Wiener index and the number of species to evaluate the diversity and richness of bacteria and microorganisms in a single sample, and Shannon index curve and dilution curve to evaluate whether the sequencing depth meets the subsequent molecular biological analysis (Zhang et al., 2015); (7) analyze the β diversity of bacteria and microorganisms among different samples by cluster analysis of the unweighted pair-group method with arithmetic means (UPGMA) method based on the PCoA and OTU level of UniFrac distance at the genus level (Lozupone and Knight, 2005); (8) predict the function of the rice wine koji group by PICRUSt (Langille et al., 2013), an open-source software of bioinformatics, and obtain the clusters of orthologous groups (COG) of each sample; (9) use the BugBase tool^1^ to classify, count, and visualize the bacterial phenotype of all koji samples.

### Statistical Analysis

Statistical analysis and data visualization were performed using R,^2^ origin (version 2017), MATLAB (version 216b), and SAS (version 8.1). The Mann–Whitney rank sum test or the Kruskal–Wallis test was used to evaluate the difference in the microbial diversity index of rice wine koji among the different groups. When the variance was uneven, the non-parametric Mann–Whitney test was used to test the significance of the difference between two or more groups of samples. On the graphs, significant differences are marked by asterisks. Based on the weighted and unweighted uniform distance, the bacterial community structure of samples from different regions was analyzed and compared by principal coordinate analysis and Euclidean distance principal co-ordinate analysis (PCoA) was used to show the differences between the bacterial groups in rice wine koji samples from different regions.

## Results and Discussion

### Sequence Coverage of Bacterial Community Across All Samples

In this study, 92 samples of rice wine koji (Hubei 31, Sichuan 30, Guangxi 31) were collected, and the bacterial diversity of rice wine koji samples from different regions was analyzed using Illumina MiSeq high-throughput sequencing technology and bioinformatics technology. In total, 4,247,804 high-quality sequences were produced in high-throughput sequencing, with 46,171 ± 8,177 (mean ± SD; range 20,897–72,651) per sample. Subsequently, 139,635 OTUs were obtained after PyNAST alignment and 100% sequence consistency clustering and chimera removal. After removing singleton OTUs, the average number of OTUs per sample was 1,518 (range: 306–2,400; SD = 472). By integrating the RDP, Greengenes, and Sliva databases, the classification level of microorganisms in representative sequences of bacterial OTU was determined. After quantifying the α diversity of each sample, the rarefaction curve showed a gradual upward trend with an increase in sequence number, whereas the Shannon index curve gradually entered a plateau when the sequence number was approximately 16,000 (Supplementary Figure 1). This indicated that with an increase in sequencing depth, there may be new OTUs and species types, but their bacterial diversity will not change significantly, indicating that the 4,247,804 sequences measured in this study were sufficient to meet the requirements of the subsequent bioinformatics analysis.

**FIGURE 1:**
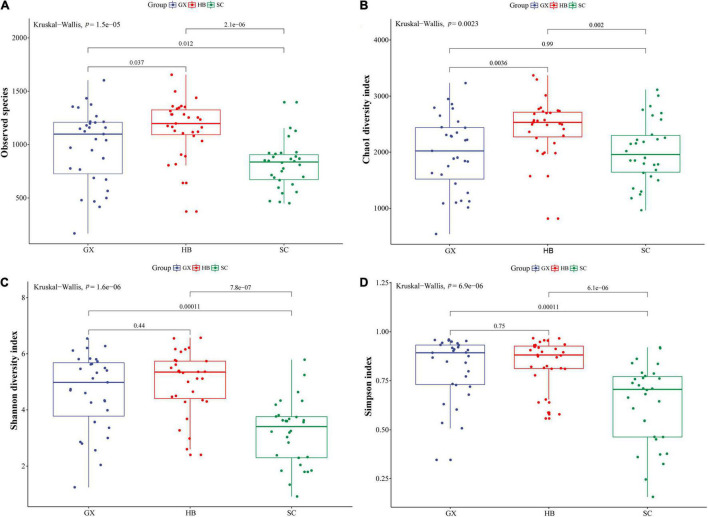
Boxplots of α-diversity indices. **(A)** Number of observed species; **(B)** Chao1 index; **(C)** Shannon index; **(D)** Simpson index. GX, HB, and SC represent Guangxi Province, Hubei Province, and Sichuan Province, respectively. The whisker caps represent the minimum and maximum values. The box plot middle, upper, and lower lines represent the median value and first and third quartiles, respectively; values were calculated under the condition of a sequencing depth of 32,010 sequences.

Bacterial community data from rice wine koji samples from different regions were analyzed and compared to investigate the differences in bacterial community. The abundance and uniformity of bacterial species were characterized by OTU rank and OTU sequence number as the abscissa and ordinate of rank abundance curve (Supplementary Figure 2). In the abscissa, the width of the rice wine koji sample curves from the three regions was different, and the width of HB and GX rice wine koji sample curves was larger than that of the SC rice wine koji sample. In the vertical direction, there was no obvious difference in the curve smoothness among samples from three regions. Therefore, the rice wine koji sample from three regions shows certain differences in species richness and relatively consistent species distribution uniformity. Using the Kruskal–Wallis test, we explored the species richness and diversity of bacteria in more detail, as well as the composition and differences of bacterial community structure among rice wine koji samples from different regions (Figure 1). Overall, the observed species and Chao1 index of the Hubei samples were the largest, followed by the Guangxi samples, and smallest in the Sichuan samples. There was a significant difference in diversity between Hubei and other regions (*p* < 0.001) (Figures 1A,B), which indicated that Hubei had the most bacterial species and the highest abundance of bacterial communities. In contrast, the Shannon index (Figure 1C) showed that the bacterial diversity of the HB and GX rice wine koji samples was higher than that of SC rice wine koji samples (*p* < 0.01), which was consistent with the results of the average number of reads, OTUs, and rank abundance curve. This indicated that the difference of the fermentation environment in different regions may be the leading factor in the different bacterial community structures in rice wine koji.

**FIGURE 2:**
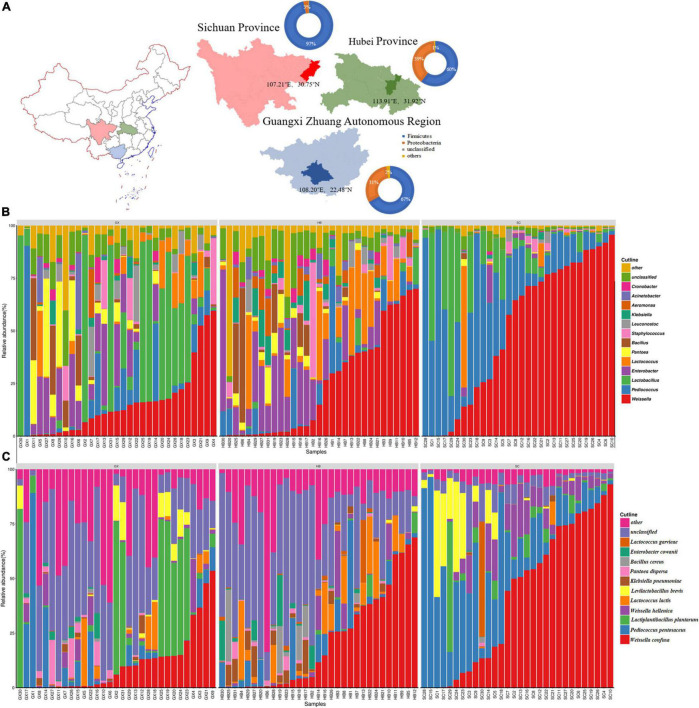
Analysis of location, α-diversity, and dominant phyla in the samples of rice wine koji from different regions. Phyla, genera, and genus with a mean relative abundance of >1.0% were defined as “dominant” and those remaining as “others.” **(A)** Location of sampling sites. The pie chart represents the relative abundance of the dominant bacterial phyla. **(B)** Histogram of relative abundance of dominant bacterial genera in samples from different regions. **(C)** Relative abundance and bacterial diversity of rice wine koji at the species level.

### Composition and Difference of Bacterial Community Structure of Rice Wine Koji Samples From Different Regions Based on Multivariate Statistical Analysis

In total, 22 phyla were identified from all rice wine koji samples; Firmicutes was the most abundant phyla with an average relative content of 74.32%, followed by Proteobacteria (24.48%); these accounted for 98.80% of the total bacterial sequence. The relative content of Firmicutes in Sichuan was the highest, at 96.42% (Figure 2A).

At the genus and species levels, 479 bacterial genera and 935 bacterial species were identified. *Weissella* was the most abundant genus (contributing to 30.14% of the total number of sequences), followed by *Pediococcus* (14.01%), *Lactobacillus* (12.68%), *Enterobacter* (8.69%), *Lactococcus* (6.31%), *Pantoea* (3.25%), *Bacillus* (3.16%), *Staphylococcus* (2.77%), *Leuconostoc* (2.03%), *Klebsiella* (1.92%), *Aeromonas* (1.43%), *Acinetobacter* (1.37%), and *Cronobacter* (1.03%) (Figure 2B). Similar to previous studies (Zhao et al., 2020), *Weissella*, *Lactobacillus*, *Lactococcus*, *Bacillus*, *Enterococcus*, and *Cronobacter* were the common dominant bacterial genera, but their abundance was quite different. In previous studies, there was no dominance of *Pediococcus*, *Pantoea*, *Staphylococcus*, *Leuconostoc*, *Aeromonas*, and *Acinetobacter*. Therefore, we speculated that the micro-ecological environment in different production seasons may affect the diversity and abundance of the community composition. To explore the influence of environmental factors on the community composition of rice wine koji, we performed an analysis of the community structure of rice wine koji produced in different seasons. At the species level, the average relative abundance of 11 types of bacteria was more than 1%, namely, *Weissella confusa* (24.51%), *Pediococcus pentosaceus* (13.43%), *Lactiplantibacillus plantarum* (7.21%), *Weissella hellenica* (4.56%), *Lactococcus lactis* (3.55%), *Levilactobacillus brevis* (3.47%), *Klebsiella pneumoniae* (1.38%), *Pantoea dispersa* (1.18%), *Bacillus cereus* (1.15%), *Enterobacter cowanii* (1.11%), and *Lactococcus garvieae* (1.06%) (Figure 2C); the aforementioned species accounted for 62.61% of the total number of reads.

**FIGURE 3:**
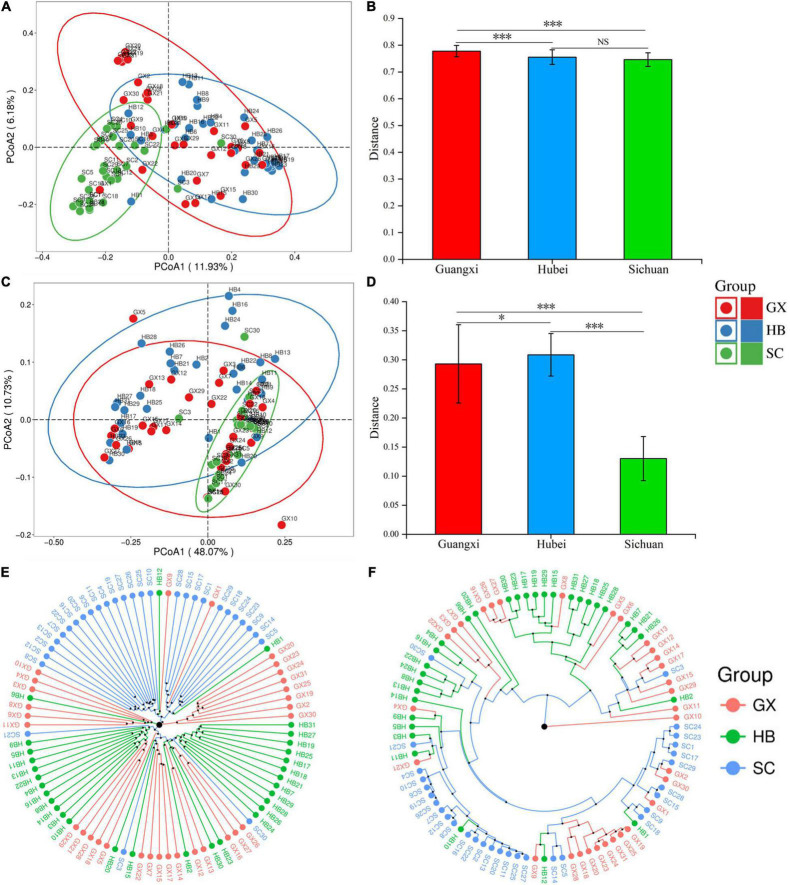
Unweighted **(A)** and weighted **(B)** PCoA score plots based on principal components 1 and 2. **(C,D)** Euclidean distance clustering analysis; clustering of rice wine koji samples based on unweighted **(E)** and weighted **(F)** UniFrac distances. Significant differences are represented by ***(0.0001 ≤ *p* < 0.001), *(0.01 ≤ *p* < 0.05), and ns (*p* ≥ 0.05).

The β diversity index was used to evaluate further differences in bacterial community composition in rice wine koji from different regions (Figure 3). Based on an unweighted PCoA, we found that although some samples overlap in spatial distribution, there was obvious clustering among regions. The SC samples were closely gathered in the lower left corner of the coordinate axis, the HB samples are mostly in the upper right corner, and the GX samples are dispersed but still mainly distributed on the negative axis of PCoA2 (Figure 3A). When considering the relative abundance of each OTU, the HB and GX samples were difficult to distinguish and were distributed throughout PCoA1, whereas the SC samples were mostly concentrated in the fourth quadrant (Figure 3B). As shown by this analysis, the bacterial community composition of rice wine koji samples was similar in areas with high overall bacterial abundance, but the composition and content were quite different when the overall bacterial abundance was low.

We then analyzed samples based on the distance matrix obtained from Unifrac analysis and unweighted and weighted Euclidean distances (Figures 3C,D). Based on unweighted one-way ANOVA (Figure 3C), the difference between the HB (0.31 ± 0.04) and SC (0.13 ± 0.04) rice koji microbial community structure groups was calculated by Euclidean distance, and the Mann–Whitney test showed that the differences between the two groups was extremely significant (*p* < 0.001) (Figure 3D). To verify this result, UPGMA was used to analyze the microbial community structure of rice wine koji in three areas. The UPGMA results were consistent with those of PCoA (Figures 3E,F). Significant differences in the bacterial community structure among rice wine koji samples from the three regions were confirmed by PERMANOVA (unweighted UniFrac distance, *p* = 0.001; weighted UniFrac distance, *p* = 0.001).

High-throughput sequencing technology can explore the microbial composition of a series of traditional fermented foods without culture and can therefore identify a large number of microorganisms that have not been isolated in culture. Using metagenomics techniques, Zhang et al. (2016) described the metabolic potential of the bacteria population and microbial community in Yucha, observed that *Lactobacillus* and *Weissella* were the main genera in Yucha, and revealed their functional characteristics in carbohydrate metabolism in the Yucha microbiota. Compared with the microbial diversity of Yucha, *Weissella*, *Lactobacillus*, and *Leuconostoc* were the dominant genera in rice wine koji. However, *Leuconostoc* was not abundant in Yucha. *Leuconostoc* is a common lactic acid bacterium with a rich enzyme system encoded by various genomic islands, which are used for fiber and polysaccharide fermentation, as reported previously (Andreevskaya et al., 2016). Rice wine koji is made from glutinous rice and various plant materials, and the micro-ecological environment is suitable for the growth of *Leuconostoc*. Therefore, *Leuconostoc* is often isolated from the fermented food of plants.

### Taxonomic Complexity of the Bacterial Communities

**FIGURE 4:**
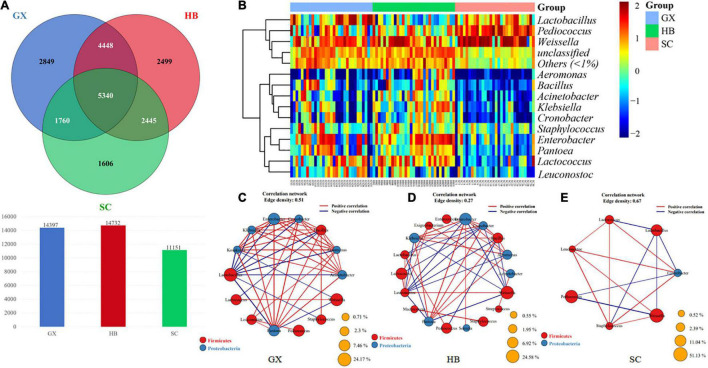
**(A)** Venn diagram showing the number of taxa shared and unique between HB, GX, and SC samples at the genus level. **(B)** Analysis of similarity among rice wine koji samples fermented in different regions based on the relative abundance of fungal OTUs (operational taxonomic units). **(C–E)** Correlation analysis of dominant bacteria.

To further identify the key groups of microorganisms in rice wine koji samples from the three regions, we investigated the differences in bacterial flora among the GX, HB, and SC samples at the genus level and in the OTUs. As shown in the Wayne diagram in Figure 4A, 5340 genera were common among the three groups; 2,849 genera were found only in GX, 2,499 genera were found only in HB, and 1,606 genera were found only in SC, accounting for 40.97, 35.94, and 23.09% of all qualified sequences after the quality control, respectively. The total number of generics in the HB group was higher than in the GX group (14,732 > 14,397). Overall, although there are many core bacterial groups in the rice wine koji samples from the three regions included in this study, some rice wine koji samples may contain some relatively low-abundance and unique bacterial species.

The microbial β diversity data indicate that there were many different OTUs in rice wine koji samples collected from different regions (Figure 4A), although *Weissella* was the dominant genus in all samples. Geographically, SC is located in the hinterland of southwest China, surrounded by mountains; HB is located in central China, with various landform types; and Guangxi is located in south China, facing the sea. Therefore, the geographical characteristics of these areas are significantly different. Indeed, the composition of microorganisms in fermented foods from different geographical areas is affected by many factors (Ehrmann et al., 2003).

The analysis of similarity among different rice wine koji samples based on the relative abundance of bacteria genus level is shown in Figure 4B; the color depth represents the relative abundance of the microorganism genus. The abundance of bacteria in samples in the HB area was relatively large, and there was a significant difference in bacterial composition between the GX and SC samples compared with the HB samples. We further identified the core bacterial groups of samples from three regions from the perspective of classification. As shown in Figures 4C–E, rice wine koji samples from GX and HB were similar in classification: Firmicutes was the dominant bacterial phylum; *Lactococcus*, *Lactiplantibacillus*, *Weissella*, and *Enterobacter* were the richest genera in the GX samples (Figure 4C); and *Weissella*, *Lactobacillus*, *Enterobacter*, and *Bacillus* were the most abundant genera in HB samples (Figure 4D). In contrast, *Pediococcus* of the Firmicutes phylum was the most abundant genus in the SC samples (Figure 4E).

LAB are considered as the main microbial flora present in a wide range of traditional fermented products. They are also the most important bacteria for food fermentation, pharmaceuticals, and special diets, and are considered as the most suitable candidates for probiotics as they are native inhibitors of the healthy human gut (Bao et al., 2012; Marco et al., 2017). In our research, *Weissella* (30.14%), *Pediococcus* (14.01%), and *Lactobacillus* (12.68%) were the dominant LAB groups in rice wine koji samples (Figure 2B). The results in this study were the same as those of Bora et al. (2016), and it is confirmed that rice wine koji contains a variety of LAB flora, such as *Lactobacillus*, *Lactococcus*, and *Weissella*. Among the dominant genera, *Lactobacillus* can convert saccharides into lactic acid during the fermentation of rice wine koji; this is accompanied by the formation of various byproducts, such as ethanol, acetic acid, and NO_2_, which not only give rice wine koji a unique flavor but also have an inhibitory effect on miscellaneous bacteria and harmful bacteria. As a heterotypic lactic acid bacterium, *Weissella*’*s* main metabolites are lactic acid and acetic acid. Studies have shown that *Weissella* can produce oligosaccharides and exopolysaccharides (mainly dextran), and that these substances can exist in fermented products for human use. In contrast to the results of this study, Lu et al. (2017) revealed that the dominant bacterial populations in rice wine koji from Yichang City and Suzhou City, China were *Aeromonas*, *Acinetobacter*, *Pseudomonas*, *Enterobacter*, *Bacillus*, and *Lactococcus*, providing further evidence that rice wine koji from different regions has unique bacterial flora. In summary, the LAB community of rice wine koji is complex and diverse, and there were differences in the LAB of rice wine koji from three different regions. In addition, 23.80% of the sequences in this study could not be classified at the genus or species level. This may be because there are many uncultured microorganisms in rice wine koji that have no specific taxonomic position in Greengenes or the Ribosomal Database Project (RDP) database, or because rice wine koji has formed a unique microbial flora owing to its special production environment and technology. It is difficult to fully capture the rice wine koji biodiversity map (Kim et al., 2015) by using only traditional isolation and identification methods. Second-generation sequencing technology effectively makes up for this shortcoming, breaking through the technical bottleneck that is the inability to separate and purify microorganisms by using culture medium, and shows the feasibility and obvious advantages of high-throughput technology in rice wine koji microbial research; moreover, it provides a new research focus for follow-up studies of the microbial species composition of traditional fermented foods.

*Klebsiella* is also one of the dominant genera in rice wine koji samples; it is an important conditional pathogen and iatrogenic infectious bacteria and accounted for 1.38% of the total bacterial sequence. This bacterium is distributed throughout nature (Kaur et al., 2018): in water, soil, and food. It is often the cause of pneumonia, bacteremia, enteritis, meningitis, and urinary tract inflammation, and severe infection may result in death (Follador et al., 2016). The presence of these bacteria results from the open production environment of rice wine koji, which can lead to pollution by livestock manure and sewage during fermentation. Therefore, identifying the pollution route of *Klebsiella* and avoiding cross-contamination are key factors for the reduction or elimination of the potential risks of this type of infection. Therefore, while improving the processing environment, actively screening for LAB with excellent fermentation characteristics for the purebred fermentation of rice wine koji should have a positive effect on the industrialization process of rice wine koji and the improvement of food safety.

Although there are few studies of the bacterial diversity of rice wine koji at present, there have been reports on microbial diversity in other traditional fermented foods in China, such as kimchi (Wang and Shao, 2018), dough (Suo et al., 2020), and fermented milks (Zhadyra et al., 2021). Their results were similar to those of this study; that is, physical chemistry (temperature, humidity, and pH value) and geographical factors (longitude, latitude, and altitude) have a more profound impact on the differences of samples in different fermentation environments. These environmental differences affect the growth, metabolism, and reproduction of the microbial community during the fermentation of rice wine koji, and have led to the difference in the bacterial community structure of rice wine koji samples from different regions. It is worth noting that this difference exists not only among rice wine koji samples from different regions but also among samples from the same region. Although the production area is the same, rice wine koji is handmade and depends completely on the experience of the producers; the production environment of each family is very extensive and there is no uniform standard. These factors also have a certain influence on the bacterial community structure of rice wine koji, causing some differences in the bacterial community structure of rice wine koji samples from the same area. As one of the commonly used starter strains in food industry, LAB strains commonly used in the market at present are mostly isolated from healthy human intestines, traditional fermented dairy products, or pickles, and their production methods and substrates are quite different from those of rice wine koji. Therefore, the use of rice wine koji as a source for new LAB strains and for actively performing the collection and preservation of LAB strains resources is of great significance for both the development and utilization of commercial LAB in China and for the industrial production of rice wine.

### Prediction of Microbiome Phenotypes and Bacterial Functional Potential in Rice Wine Koji Samples

**FIGURE 5:**
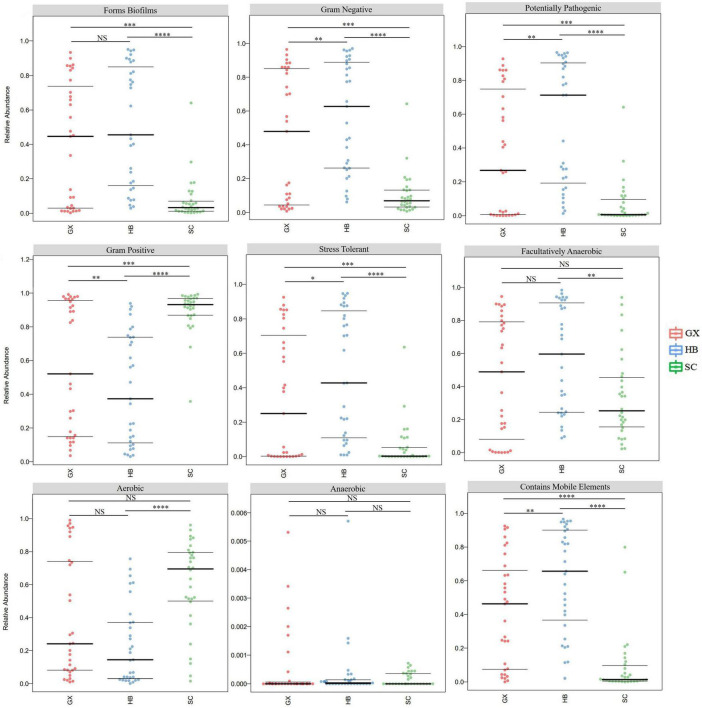
Comparative analysis of bacterial phenotypic results of rice wine koji samples. Significant differences are represented by ****(*p* < 0.0001), ***(0.0001 ≤ *p* < 0.001), **(0.001 ≤ *p* < 0.01), *(0.01 ≤ *p* < 0.05), and ns (*p* ≥ 0.05). HB, GX, and SC represent Hubei City, Guangxi Province, and Sichuan Province, respectively.

As shown by the potential prediction of phenotypic function in the bacterial community in rice wine koji samples from different regions, nine potential microbial phenotypes were detected, including biofilm forming, gram negative, potentially pathogenic, gram positive, and stress tolerant (Figure 5). The differences in abundances of the eight phenotypic functions (biofilm forming, gram negative, potentially pathogenic, gram positive, stress tolerant, artificially anaerobic, aeronautical, and mobile element containing) were significant (*p* < 0.05), which indicated that the bacterial communities with these functions were affected by the producing region. However, there was no significant difference in the anaerobic function of rice wine koji samples (*p* > 0.05). This may be due to the fact that rice wine koji is cultivated and produced in an open system in a specific ecological environment. Consequently, there was no significant difference in the anaerobic function of rice wine koji samples from different regions (*p* > 0.05).

It is worth noting that in four functions (biofilm forming, gram negative, potentially pathogenic, and stress tolerant), the microorganisms in rice wine koji samples from the three regions showed the same order of functional abundance (HB > GX > SC), whereas in the gram-positive function, the opposite trend occurred (HB < GX < SC). Biofilms are a complex community of microorganisms attached to the surface or related to interface, and are usually composed of many species that interact with each other and the environment (Davey and O’toole, 2000). The existence of biofilm as a barrier creates a stable internal environment for the bacterial community and may also exert bacterial antagonist activity to prevent potential pathogens. Similarly, biofilm formation can also promote pathogenicity, thus improving stress tolerance (Mamphogoro et al., 2020). This may explain the same trends in biofilm formation, potential pathogenicity, and stress tolerance in bacterial communities of rice wine koji samples from different regions. In addition, *Weissella*, *Lactobacillus*, *Enterobacter*, *Bacillus*, and *Acinetobacter* were the absolute dominant bacterial genera in rice wine koji samples. It is known that these genera have antagonistic effects on fungal pathogens, and can reduce cucumber Fusarium wilt, *Fusarium oxysporum*, and other fungal pathogens, and stimulate the growth of other vegetables and fruit crops, including chickpeas and cucumbers (Mata et al., 2013; Wachowska et al., 2013; Wang et al., 2016; Palmieri et al., 2017). Therefore, if biological and chemical methods of pest control are recommended, these antagonists can be recommended for integrated pest management (IPM) programs (Gangwar, 2017). In addition, *Lactobacillus* is a group of gram-positive bacteria that can ferment glucose and lactose into lactic acid, which is beneficial to human health (Wagar et al., 2009). They produce lactic acid in the process of proliferation, forming an acidic environment that inhibits the growth of other bacteria, which leads to the opposite trends in gram-negative and gram-positive functions.

It is worth mentioning that although there were a similar number of pathogenic bacteria in rice wine koji from the three regions, the pathogenic potential of bacteria in the SC region was significantly lower than that in the HB and GX regions (*p* < 0.001). The method of making rice wine koji used by farmers is more traditional, and the rice wine koji is more open to the environment. Pathogens or other microorganisms in the environment may cause the pollution of rice wine koji. Therefore, a significant positive effect would be gained by standardizing the product method of making rice wine koji, maintaining environmental hygiene, and screening for fermentation strains with excellent characteristics to improve the quality and safety of rice wine.

To better understand the important role of the microbial community in rice wine koji samples, the data from the Cluster of Orthologous Groups (COG) database were further analyzed using the PICRUSt program based on high-throughput sequencing data of 16S rRNA. As a result, 4,712 types of functional protein or enzyme genes belonging to 23 types of functional genes were identified, and the COG map of bacteria was obtained.

**FIGURE 6:**
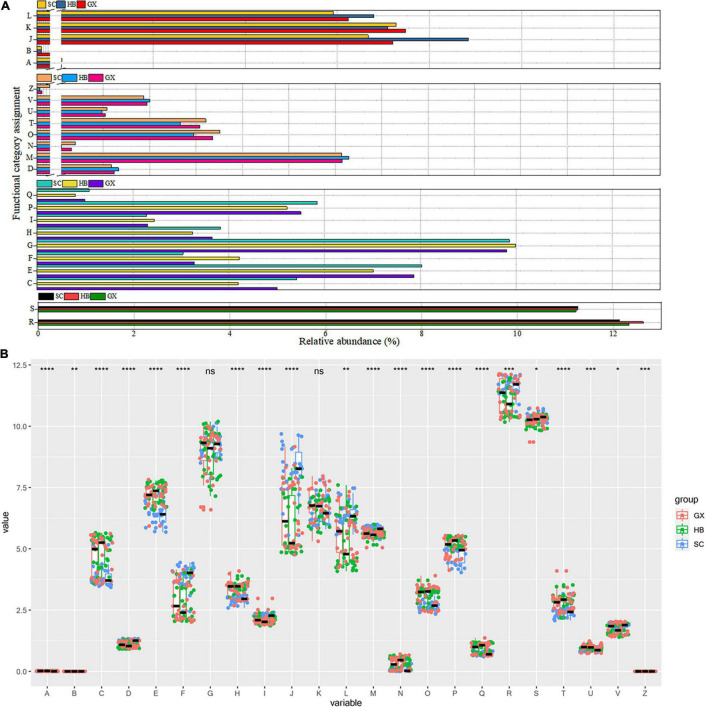
Overview of the relative abundance of the bacterial profile **(A)**. Correlation analysis of COG functional categories of rice wine koji samples between regions **(B)**. COG functional categories codes are as follows: A = RNA processing and modification; B = chromatin structure and dynamics; C = energy production and conversion; D = cell cycle control, cell division, chromosome partitioning; E = amino acid transport and metabolism; F = nucleotide transport and metabolism; G = carbohydrate transport and metabolism; H = coenzyme transport and metabolism; I = lipid transport and metabolism; J = translation, ribosomal structure, and biogenesis; K = transcription; L = replication, recombination, and repair; M = cell wall/membrane/envelope biogenesis; N = cell motility; O = posttranslational modification, protein turnover, chaperones; P = inorganic ion transport and metabolism; Q = secondary metabolites biosynthesis, transport and catabolism; R = general function prediction only; S = function unknown; T = signal transduction mechanisms; U = intracellular trafficking, secretion, and vesicular transport; V = defense mechanisms; Z = cytoskeleton. Significant differences are represented by ****(*p* < 0.0001), ***(0.0001 ≤ *p* < 0.001), **(0.001 ≤ *p* < 0.01), *(0.01 ≤ *p* < 0.05), and ns (*p* ≥ 0.05).

Enrichment of metabolic function is observed in Figure 6A, indicating the vigorous microbial metabolism in the rice wine koji samples. Among these metabolic functional characteristics, the relative abundance of G (carbohydrate transport and metabolism), E (amino acid transport and metabolism), and P (inorganic ion transport and metabolism) functional categories in all rice wine koji samples was significantly higher than that of other metabolic functional categories (*p* < 0.001) (Figure 6B). As expected, most of the sequences were functionally assigned to genes related to energy metabolism, carbohydrate metabolism, and ion transporters, indicating that the bacterial species in rice wine koji samples were efficient in the utilization of protein and carbohydrate. However, some differences in functional gene abundance observed between the different regions may be more directly related to the unique geographical conditions found in the different regions, including water availability and plant biomass. Similarly, frequent water stress can explain why the relative abundance of genes related to class E (amino acid transport and metabolism) metabolic function was high: bacteria usually use amino acid–based solutes for osmotic regulation. Inorganic ions are necessary for basic bacterial metabolism because these ions act as cofactors of enzymes and promote many biochemical reactions. High expression of genes related to P (inorganic ion transport and metabolism) and C (energy production and conversion) functional categories can drive the energy demand of bacterial flora, and the energy production of microbial flora mainly depends on the phosphorylation of substrate level through sugar fermentation to acetate. At the same time, we also found that the overexpression of COGs [C (energy production and conversion), O (posttranslational modification, protein turnover, chaperones), and T (signal transduction mechanism)] involved in cell movement may indicate the strengthening of nutrition-seeking activity driven by microorganisms in rice wine koji samples, which may increase the signal sensing ability of microorganisms and enable them to make better use of nutrients. The four cognitive functional categories (cellular processes and signaling, information storage and processing, metabolism, poorly characterized) are very important for the fermentation of rice wine koji. The upregulation of this gene category indicates the function of bacteria in improving the fermentation efficiency of rice wine koji, shortening the production cycle, and improving the utilization rate of raw materials.

In the production process of fermented products, microorganisms transform raw materials into products with higher value, usually by prolonging the shelf-life of raw materials and improving the production of sensory characteristics to increase the nutritional value of products (Von Mollendorff et al., 2006; Das et al., 2012; Cai et al., 2020). At a functional level, Zhang et al. (2016) focused on the microbial metabolic pathways of carbohydrates in Yucha from different regions. In the rice wine koji samples, other than carbohydrate metabolism, the amino acid metabolism (Figure 6A) remained at a high level because of the high protein content of glutinous rice. Therefore, not only the region but also the raw materials used may play a role in the formation of food microbiota.

## Conclusion

In this study, the bacterial composition of rice wine koji samples collected from Hubei Province (HB), Sichuan Province (SC), and Guangxi Province (GX) in China was studied in depth and characterized. Firmicutes and Proteobacteria were the dominant phyla; *Weissella*, *Pediococcus*, *Lactobacillus*, *Enterobacter*, *Lactococcus*, *Pantoea*, *Bacillus*, *Staphylococcus*, *Leuconostoc*, *Klebsiella*, *Aeromonas*, *Acinetobacter*, and *Cronobacter* were the main strains in all the rice wine koji samples. The bacterial community structure of rice wine koji samples from different production areas was significantly different (*p* < 0.05). HB and GX rice wine koji were similar in composition, and their representative bacteria were lactic acid bacteria. In addition, eight of the nine predicted phenotypic functions were significantly different among different regions (*p* < 0.05), and bacterial metabolism was vigorous. The results provide theoretical guidance for improving the preparation technology and quality of rice wine koji.

It is worth mentioning that although MiSeq sequencing technology makes up for the shortcomings of low flux, limited resolution, and the small size of the separated fragments, it has its own shortcomings, including short sequencing reading, so it is impossible to judge whether they have played an active role in the fermentation of rice wine koji. Therefore, the purpose of this study was twofold. First, the analysis of the microbial community structure of rice wine koji in this study, the separation, identification, and screening of the dominant microorganisms, and the pure culture and compound of strains with excellent fermentation characteristics may allow the development of a special starter for rice wine, which may be of great significance to the improvement of food safety and industrial development of rice wine. Second, it has laid the foundations for a study of the diversity and community structure of microorganisms in rice wine koji by using metagenome technology, and to explore its potential functions and relationship with environment and product quality. As one of the commonly used starter strains in the food industry, it will be of great significance to use rice wine koji as a source of new LAB strains and actively perform the collection and preservation of LAB strain resources, both for the development and utilization of commercial LAB and for enhancing the industrial production of rice wine.

## Data Availability Statement

The datasets presented in this study can be found in online repositories. The names of the repository/repositories and accession number(s) can be found below, NCBI; PRJNA760782.

## Author Contributions

XZ: formal analysis, writing—original draft, writing—review and editing, and visualization. FX: data curation and visualization. FT: methodology and project administration. WC: data curation. ZG: software, investigation, and resources. QH: validation. WS: data curation. CS: conceptualization, supervision, and funding acquisition. All authors contributed to the article and approved the submitted version.

## Conflict of Interest

The authors declare that the research was conducted in the absence of any commercial or financial relationships that could be construed as a potential conflict of interest.

## Publisher’s Note

All claims expressed in this article are solely those of the authors and do not necessarily represent those of their affiliated organizations, or those of the publisher, the editors and the reviewers. Any product that may be evaluated in this article, or claim that may be made by its manufacturer, is not guaranteed or endorsed by the publisher.

## Footnotes

1
https://bugbase.cs.umn.edu/

2
http://www.r-project.org/
