# Supplementary figures and images for: Bacterial Communities and Prediction of Microbial Metabolic Pathway in Rice Wine Koji From Different Regions in China
Source: Front Microbiol. 2022 Jan 3;12:748779. doi: 10.3389/fmicb.2021.748779 (PMC8762310; doi:10.3389/fmicb.2021.748779)

**Figure captions**

**Fig. S1** The Sparse curve (A) and Shannon index curve (B)

**Fig. S2** OTU Rank curve

**Fig. S1**


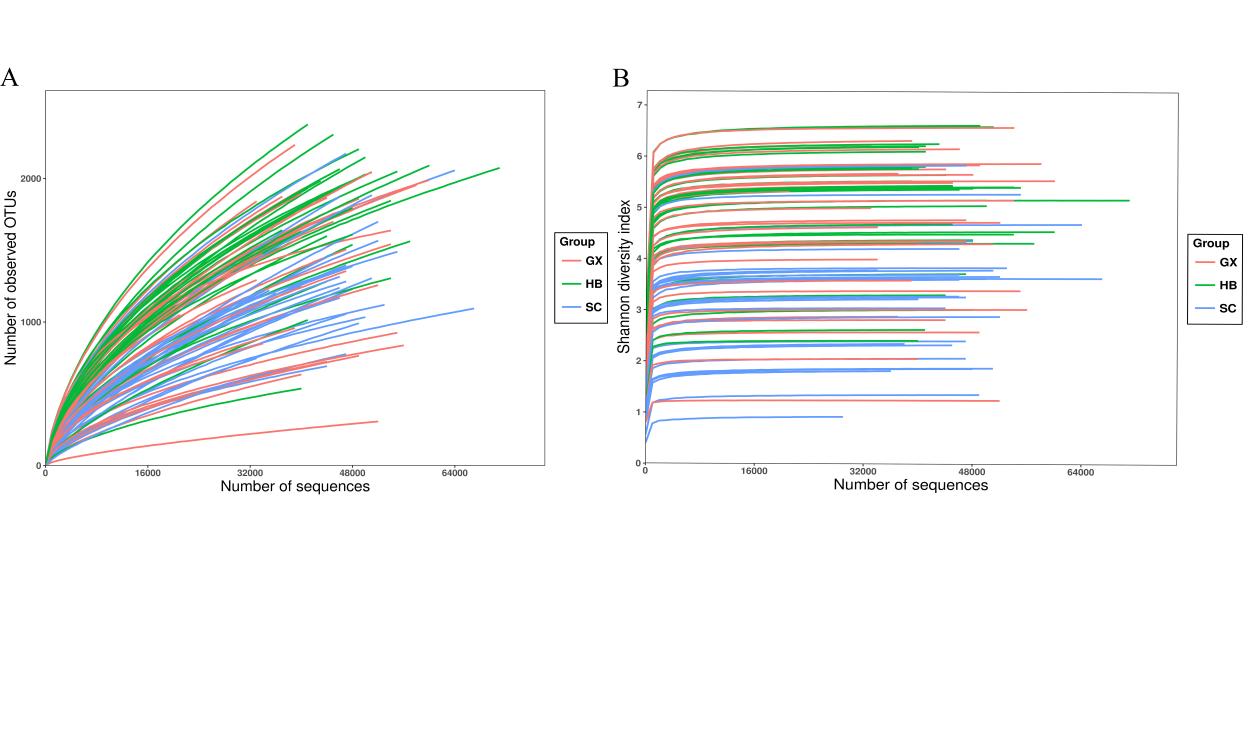


**Fig. S2**


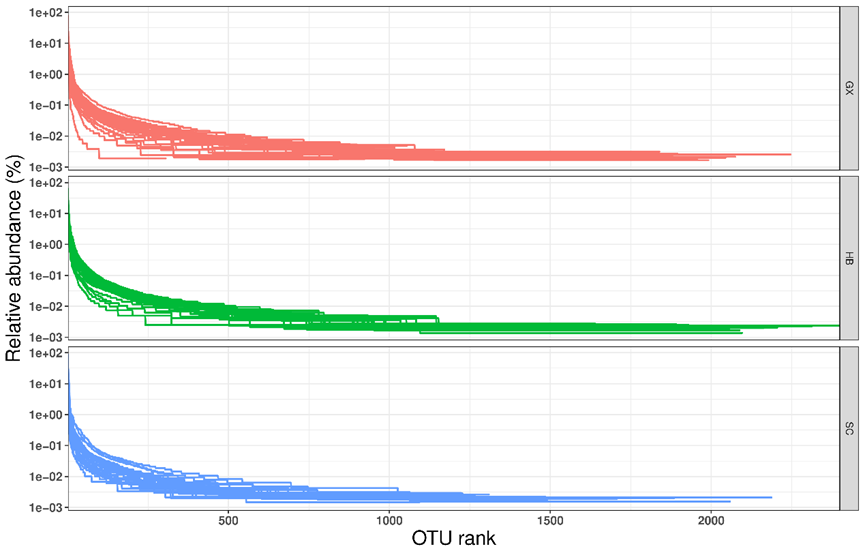

Supplement: Supplementary file 1 [file Data_Sheet_1.docx]
